# Photocuring of Epoxidized Cardanol for Biobased Composites with Microfibrillated Cellulose

**DOI:** 10.3390/molecules24213858

**Published:** 2019-10-25

**Authors:** Sara Dalle Vacche, Alessandra Vitale, Roberta Bongiovanni

**Affiliations:** Department of Applied Science and Technology, Politecnico di Torino, Corso Duca degli Abruzzi 24, 10129 Torino, Italy; alessandra.vitale@polito.it (A.V.); roberta.bongiovanni@polito.it (R.B.)

**Keywords:** biobased epoxy, cardanol, cationic photocuring, microfibrillated cellulose, biobased composites, sustainable materials

## Abstract

Cardanol is a natural alkylphenolic compound derived from Cashew NutShell Liquid (CNSL), a non-food annually renewable raw material extracted from cashew nutshells. In the quest for sustainable materials, the curing of biobased monomers and prepolymers with environmentally friendly processes attracts increasing interest. Photopolymerization is considered to be a green technology owing to low energy requirements, room temperature operation with high reaction rates, and absence of solvents. In this work, we study the photocuring of a commercially available epoxidized cardanol, and explore its use in combination with microfibrillated cellulose (MFC) for the fabrication of fully biobased composites. Wet MFC mats were prepared by filtration, and then impregnated with the resin. The impregnated mats were then irradiated with ultraviolet (UV) light. Fourier Transform InfraRed (FT-IR) spectroscopy was used to investigate the photocuring of the epoxidized cardanol, and of the composites. The thermomechanical properties of the composites were assessed by thermogravimetric analysis, differential scanning calorimetry, and dynamic mechanical analysis. We confirmed that fully cured composites could be obtained, although a high photoinitiator concentration was needed, possibly due to a side reaction of the photoinitiator with MFC.

## 1. Introduction

The growing environmental concerns and the foreseen depletion of fossil resources call for a more sustainable economy; this gives impulse to the development of materials derived from biomass, produced with environmentally friendly processes. The photo-induced curing of biobased monomers, although still scarcely documented, is particularly suited for the preparation of sustainable polymer-based materials [1,2]: in fact, photopolymerization is considered a green technology owing to low energy requirements, room temperature operation with high reaction rates, and no need for solvents [3].

Cardanol is an alkylphenolic compound derived from Cashew NutShell Liquid (CNSL), an annually renewable and non-edible raw material extracted from cashew nutshells, byproduct of the food industry. Epoxidized and acrylated derivatives of cardanol, which may be suitable for photopolymerization, have been recently synthesized [4,5,6,7,8,9]. Commercial epoxidized cardanol resins are available on the market, and their thermal curing with amine hardeners has been reported, the thermomechanical properties obtained varying widely depending on the hardener used [10,11]. Few studies though have been published on the photo-induced cationic polymerization of epoxidized cardanol derivatives, namely using electron beam and UV (254 nm) as radiation sources. Different epoxidized derivatives of cardanol, including also epoxide groups on the aliphatic chain, have been used in these works, where hexafluorophosphate and hexafluoroantimonate type cationic initiators were employed [12,13,14]. Photopolymerized epoxy cardanol showed however relatively weak mechanical properties, due to the low functionality of the monomers, leading to a low degree of crosslinking, and to the presence of the flexible aliphatic side chain. Therefore, its use as an additive has also been explored; as an example, cationic photopolymerization with an ultraviolet source in the UVA range (315–400 nm) of a cycloaliphatic epoxy-based materials containing 10 wt.% epoxidized cardanol was reported [15]. 

UV-curing biobased monomers in combination with natural reinforcements, for fully biobased composite materials, is another interesting possibility. Microfibrillated cellulose (MFC) may be derived by a variety of wood and annual plants biomass; MFC sheets have high mechanical and barrier properties which are very attractive for packaging and electronics applications. However, these excellent properties are highly affected by humidity. Therefore, MFC has been combined with crosslinked polymer matrices. A solvent exchange process has been reported for the fabrication of composites with microfibrillated cellulose to preserve a structure of individual nanofibers; composites with a thermally cured epoxy matrix, and UV curable composites with an acrylate matrix have been prepared [16,17]. To the best of our knowledge no reports are available at the moment on composites of microfibrillated cellulose with a biobased cardanol epoxy photocrosslinked matrix.

In this work we confirm the feasibility of the photo-induced curing with UV light of a commercial epoxidized cardanol with an iodonium hexafluorophosphate type cationic initiator. The results obtained by photopolymerization are compared with those obtained for the thermal curing of an epoxidized cardanol with the same molecular structure [10]. Although the structures of the monomers and prepolymers used in previous works on photo-induced curing of epoxidized cardanol derivatives [12,13,14] are different from the one used here, some comparisons are possible and will be discussed. We hereby also confirm the possibility of preparing fully biobased photocured composites of epoxidized cardanol with microfibrillated cellulose, although a fairly high concentration of photoinitiator, of as much as 15 wt.%, was needed for leading the crosslinking reaction to completion. The reason for this is attributed to a side reaction of the photoinitiator with the microfibrillated cellulose.

## 2. Materials and Methods

### 2.1. Materials

**Figure 1:**
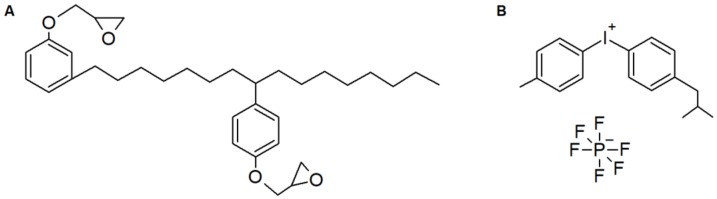
Structures of the epoxidized cardanol NC-514S (**A**) and of the photoinitiator (**B**).

The epoxidized cardanol (EC) resin NC-514S, with an epoxy equivalent weight of 418 g/mol, whose structure is shown in Figure 1, was provided by the Cardolite Corp. (US). Omnicat 250, which is a 75% solution of iodonium, (4-methylphenyl) [4-(2-methylpropyl) phenyl]-hexafluorophosphate(1-) in propylene carbonate (IGM Resins, US), was used as photoinitiator (PI); its structure is also shown in Figure 1. The microfibrillated cellulose Exilva F01-V, in the form of a paste with 10 wt.% concentration of cellulose microfibrils in water, was provided by Borregaard (Norway). For solvent exchange, acetone ≥99.5%, by Sigma-Aldrich S.r.l. (Italy), was used.

### 2.2. Preparation of the Resin and of the Composites

The photocurable resins were prepared by mixing the EC with the desired amount of photoinitiator PI, and briefly stirring with a magnetic stirrer. The resins were kept in the dark and refrigerated, and used within short time from their preparation; they were used as prepared for the resin photopolymerization study, or diluted at a 30 wt.% concentration in acetone for impregnation of MFC mats.

For the preparation of the MFC mats, the cellulose microfibrils were diluted in distilled water at 0.75 wt.% concentration, and dispersed with a homogenizer (Ultraturrax T10, IKA^®^-Werke GmbH & Co. KG, Germany) at about 20 k rpm, for 3 min. The obtained suspension was then filtered with a Büchner funnel connected to vacuum, equipped with a 47 mm diameter Durapore^®^ membrane filter made of hydrophilic poly(vinylidene fluoride) (PVDF) with 0.65 µm pore size. The microfibrillated cellulose thus formed a wet mat on the filter, containing approximately 85 wt.% of water. The water impregnating the MFC mat was then exchanged with acetone by soaking the mat in an acetone bath in a Petri dish, covered with a lid sealed with aluminum foil to prevent solvent evaporation. The acetone was refreshed three times over 24 h. To obtain dry MFC mats, used as a reference, acetone was replaced a fourth time, and was then allowed to slowly evaporate at room temperature with the lid of the Petri dish in place but not sealed. To obtain the composites, instead, once taken off the third acetone bath, the mat was directly soaked in the resin solution in acetone for 3 additional hours. The impregnated mat was then taken off the resin bath and dried under vacuum for 10 min, and then immediately irradiated with UV light. The final resin/microfibrillated cellulose weight ratio was controlled by the concentration of the resin in the acetone solution used for the impregnation of the MFC mat. An approximate resin content of 80 wt.% in the final composite was obtained when an acetone solution with 30 wt.% resin concentration was used as an impregnation bath.

A 5000-EC UV flood lamp system (Dymax Corporation, Torrington, CT, USA) with medium intensity mercury bulb (320–390 nm) was used for curing the resins, in the form of 10–50 μm-thick films on silicon wafers or glass slides, and the composites, in the form of 180 μm-thick self-standing films. The intensity was tuned at 100 ± 10 mW cm^−2^ by changing the distance between the specimen and the light source, and was measured by means of a UV Power Puck II radiometer (EIT, LLC., Leesburg, USA). The resin films were irradiated only on the free side, while the irradiation of the composites was carried out by turning the sample upside down every minute, to have homogeneous irradiation on the two sides; therefore, the irradiation time for the composites is indicated as 2 × n minutes, where n is the number of minutes of irradiation per side.

In what follows the mixtures of EC and PI are referred to as “EC-X%PI” resins, where X indicates the PI weight percent in the resin mixture, and the corresponding composites are indicated as MFC-EC-X%PI.

### 2.3. Characterization Methods

Optical microscopy was performed in in reflection and transmission mode with a Nikon SMZ18 stereo microscope (Nikon Instruments Europe B.V, The Netherlands), and in reflection dark field mode with an Olympus BX53M microscope (Olympus Italia S.R.L., Italy).

Fourier Transform Infrared (FT-IR) analysis was performed with a Nicolet iS50 spectrometer (Thermo Fisher Scientific Inc., Waltham, MA, USA). For monitoring the photocuring reaction, the resins were spread on a silicon wafer with a 10 μm wire wound bar, and were analyzed in transmission mode, in the 400–4000 cm^−1^ range, with 32 scans and a resolution of 4 cm^−1^. The dry MFC mat and the composites were analyzed by Attenuated Total Reflectance Fourier Transform Infrared (ATR FT-IR) spectroscopy analyses with an accessory equipped with a diamond crystal. The spectra were acquired in the 525–4000 cm^−1^ range, 32 scans per spectrum and a resolution of 4 cm^−1^.

The insoluble fraction of the cured resins was assessed by measuring the weight of cured films, detached from the substrate and wrapped in a fine metallic mesh, before and after immersion in toluene or acetone for 24 h, and evaporation of the residual solvent at room temperature for 24 h followed by drying at 100 °C for 1 h.

Thermogravimetric analysis (TGA) was performed using a TGA/SDTA 851e apparatus by Mettler Toledo (Switzerland). Scans were made from 25 °C to 800 °C with a heating rate of 10 °C min^−1^, under a 60 mL min^−1^ N_2_ flux, to prevent thermo-oxidative processes. The first derivative of the weight profile was calculated to better resolve the main degradation steps of the analyzed materials.

Differential scanning calorimetry (DSC) was carried out using a DSC1 STARe instrument (Mettler Toledo, Switzerland) in the temperature range from −70 °C to 180 °C, using a heating rate of 10 °C min^−1^, under N_2_ flux.

Dynamic mechanical analysis (DMA) was performed with a TTDMA (Triton Technology Ltd., Keyworth, UK) equipment, in the tensile mode. The temperature increased from −100 °C to 180 °C at a 3 °C min^−1^ rate. The frequency was set at 1 Hz, and the strain was set at 0.1% for the composites, and 0.01% for the dry MFC mat. The specimens had a length of 10 mm between the clamps, and a width of 6 to 8 mm.

Wettability measurements were performed with a FTA 1000 C contact angle meter (First Ten Ångstroms, Portsmouth, VA, USA), equipped with video camera and image analysis software (FTA32 Software version 2.1, First Ten Ångstroms, Portsmouth, VA, USA), with the sessile drop technique. The testing liquid was water (γ =72 mN m^−1^).

The water uptake was measured by immersing a dried specimen of known weight in distilled water at room temperature for 4 weeks and then weighing it again after lightly wiping the surface to remove excess water.

## 3. Results and Discussion

### 3.1. Morhology and Photocuring Behaviour

In this section, we report the photocuring of a commercial EC resin, with a cationic hexafluorophospate photoinitiator, and of its composites with microfibrillated cellulose. Dry microfibrillated cellulose mats prepared by solvent exchange, but without addition of resin, were also characterized for reference. As detailed below, while the resin containing 5 wt.% PI could reach full conversion within a few minutes of exposure to UV light, for the composites the curing reaction was somewhat hindered, and only by highly increasing the PI amount up to 15 wt.% a full cure could be obtained.

**Figure 2:**
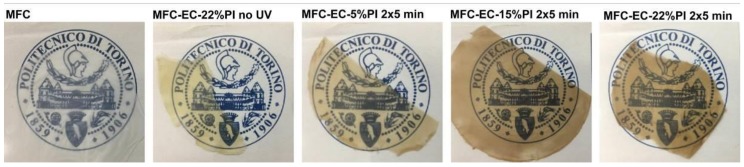
Photos of dry MFC mat, MFC-EC-22%PI composite before irradiation, and of composites irradiated for 2 × 5 min: MFC-EC-5%PI, MFC-EC-15%PI and MFC-EC-22%PI.

Photos of the dry MFC mat and of the cured composites are shown in Figure 2. The dry MFC mat obtained by solvent exchange was transparent, provided that a slow evaporation rate of acetone was ensured by keeping the Petri dish covered with his lid during drying. Otherwise, with fast evaporation rates a white non-transparent film was obtained (Figure S1); therefore, one can suggest that the porosity left in the mat upon evaporation depends highly on the solvent evaporation rate. The composites were relatively transparent; when uncured their color was yellowish, due to the color of the resin, and turned brownish upon curing, being darker with increasing PI concentration. Reaction of MFC with PI contributed to the color change, as also MFC impregnated with only PI was found to develop a brownish color upon irradiation (Figure S2).

**Figure 3:**
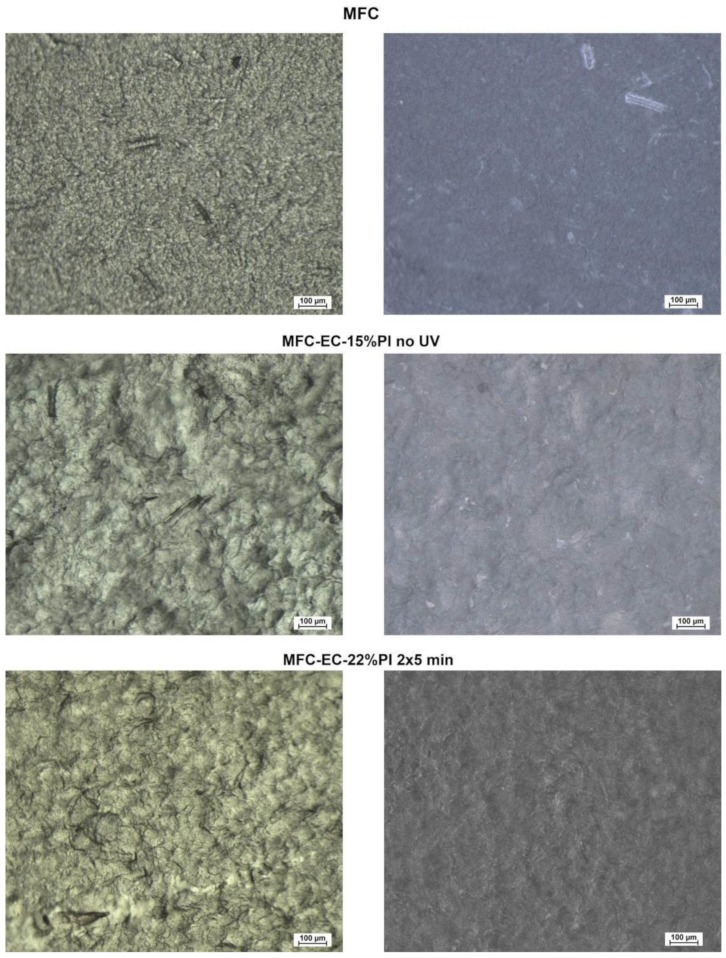
Bright field transmission (left) and reflection (right) images of: dry MFC mat, MFC-EC-15%PI before UV exposure, and MFC-EC-22%PI with 5 min of UV exposure per side.

**Figure 4:**
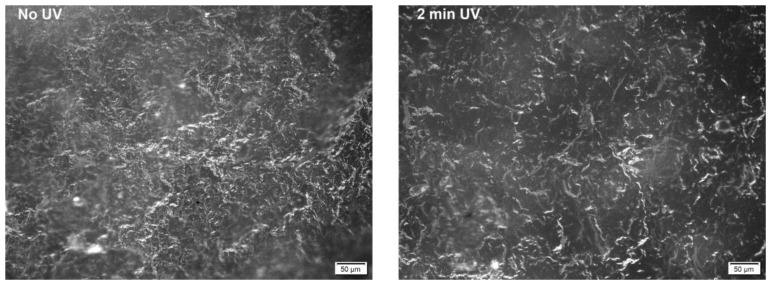
Reflection dark field images of MFC-EC-5%PI uncured (left) and with 2 min of UV exposure per side (right).

The dry MFC mat, and the uncured and cured composites were also observed by optical microscopy. Bright field images in both reflection and transmission mode are shown in Figure 3 and dark field images in reflection mode are shown in Figure 4. The homogeneous structure of entangled fibers shown by the dry MFC mat was maintained also in the composites, before and after curing. A few larger fibers were also visible. A rough surface structure, due to the cellulose microfibrils was highlighted by the images taken in reflection mode.

**Figure 5:**
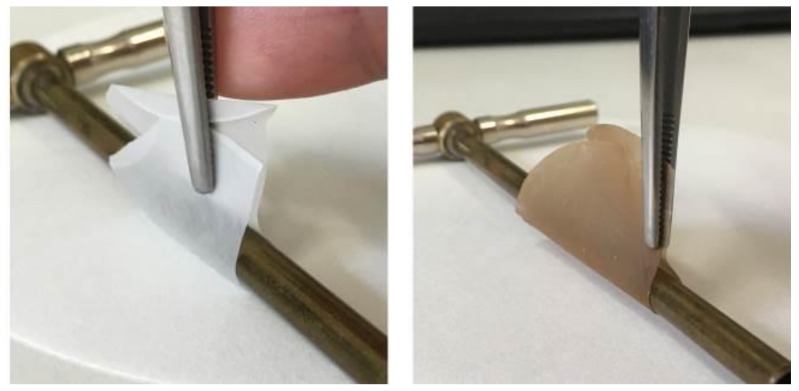
Dry MFC mat (left) and fully cured composite (right) rolled on a 4.9 mm diameter cylinder.

Both the dry MFC mat and the composites were flexible, and it was possible to roll them on a 4.9 mm diameter cylinder, as shown in Figure 5.

**Figure 6:**
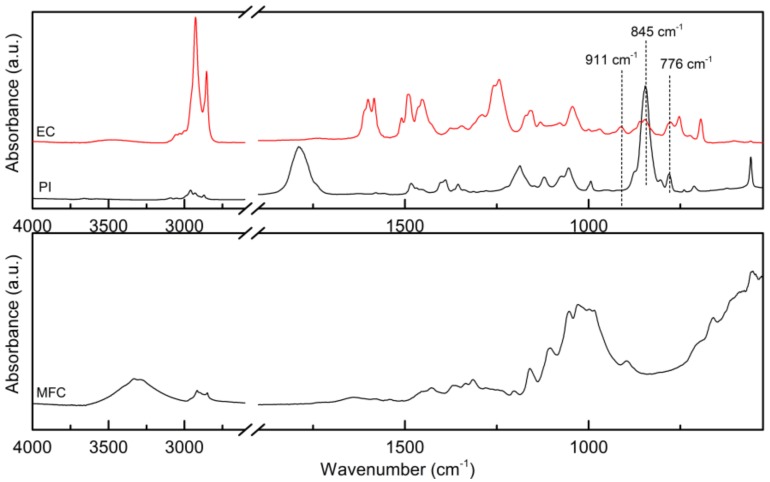
FT-IR transmission spectra of the epoxidized cardanol (EC) and of the photoinitiator (PI), and ATR FT-IR spectrum of the non-impregnated MFC mat (dry MFC).

To explore the photo-induced curing of the cardanol resin and its composites, the FT-IR transmission spectra of the EC resin and of the PI, as well as the ATR FT-IR spectrum of the dry MFC mat were recorded (Figure 6) as reference.

The spectrum of the EC resin showed two intense peaks at 2927 and 2854 cm^−1^ corresponding to sp^3^ C–H bond vibrations in the aliphatic chain of the epoxidized cardanol [4,5,12]. At higher wavenumber, some small peaks appeared; the one at 3056 cm^−1^ can be attributed to the stretching of the C–H bond of the methyl group attached to the epoxide ring [18,19]. As expected, the broad peak centered around 3350 cm^−1^ corresponding to the phenolic OH group of cardanol before epoxidation [5,20] was not present in the spectrum of EC, confirming that the hydroxyl groups are quantitatively replaced by epoxide groups. Also, the peak at about 3007–3009 cm^−1^ characteristic of C=C bonds in the aliphatic chain of cardanol [5,20] was not detectable in EC, indicating that the double bond content in this epoxidized cardanol is rather low [10]. The vibrations of the C=C aromatic bond were seen in the 1550–1650 cm^−1^ and 1400–1510 cm^−1^ regions [4,5,20]. A shoulder peak at 1258 cm^−1^ indicated the presence of the phenolic ether linkage [14,20]. The characteristic vibrations of the epoxide ring were found at 911 cm^−1^ [4,5,11,14,20], 860 cm^−1^ [4,12] and 776 cm^−1^ [4]. Among these, the most suitable to be followed to assess the degree of curing of the EC was the 911 cm^−1^ signal of the epoxide group [14], as the signals at 860 and 776 cm^−1^ overlapped with peaks characteristic of the meta substituted (670–710, 750–805, 870–900 cm^−1^) and para substituted (845 cm^−1^ γCH) aromatic rings in the EC molecule [21,22,23], and with some photoinitiator’s signals (845 cm^−1^ and 780 cm^−1^).

Indeed, the spectrum of the photoinitiator showed peaks at 1790–1800 cm^−1^ and at 780 cm^−1^, related to the propylene carbonate solvent, an intense peak at 845 cm^−1^ (stretching vibrations of the PF_6_ anion), with shoulders at 876 cm^−1^ and 804 cm^−1^, while the δ(PF_6_) bending vibrations was observed as a narrow strong band at 558 cm^−1^ [24].

The spectrum of the MFC mat was characterized by a broad O–H stretching signal in the 3500–3000 cm^−1^ region, relatively weak C-H stretching peaks at 3000–2800 cm^−1^, and in the fingerprint region intense bands attributed to the C–O stretching of the pyranose ring skeletal vibration in the 1150–1030 cm^−1^ range, and to the β-glycosidic bond vibration at 896 cm^−1^; a weak and broad peak centered at 1639 cm^−1^ reflected the presence of water adsorbed into the cellulose fibrils [25,26,27,28,29,30].

**Figure 7:**
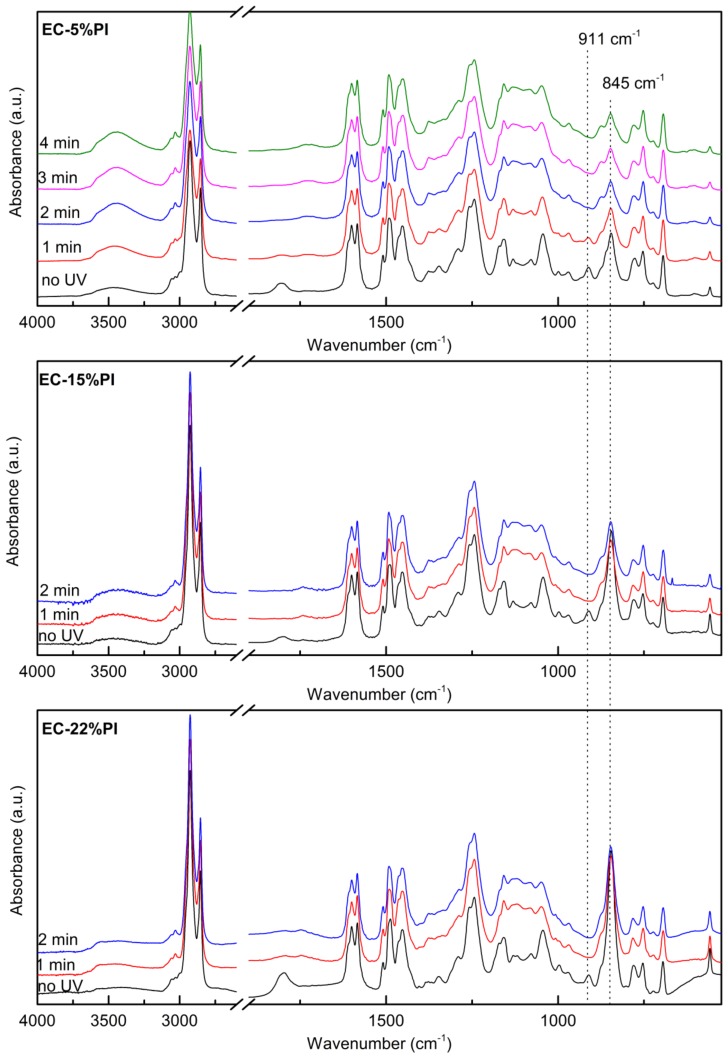
FT-IR spectra of the EC-5%PI, EC15% PI and EC-22%PI resins before and after different times of exposure to UV radiation with a 100 mW cm^−2^ intensity.

The photocuring of the resin mixtures containing 5%, 15% and 22% photoinitiator was followed; the higher amounts of photoinitiator were used also for the resin as a comparison with the composites, which could not be cured with the lowest concentration. The formulations were exposed to UV radiation at 100 mW cm^−2^ with a medium intensity mercury bulb for intervals of 1 min, and the photo-induced reaction of ring opening photopolymerization was followed by FT-IR in transmission mode (Figure 7). The peak at 911 cm^−1^, characteristic of the epoxide ring, decreased with increasing exposure time and eventually completely disappeared meaning the full conversion of the epoxide rings: for the EC-5%PI resin the peak disappeared within 3 min, while for the EC-15%PI and EC-22%PI resins less than 1 min of irradiation was sufficient. The peak at 3056 cm^−1^ and the shoulder at 860 cm^−1^ decreased as well with irradiation dose, although quantification was not possible due to overlap with other peaks. The peaks characteristic of the photoinitiator, i.e., 1800 cm^−1^, 845 cm^−1^, 780 cm^−1^ also decreased with increasing UV exposure. While the epoxide conversion proceeded, the broad peak centered around 3450 cm^−1^ increased, as expected from the formation of hydroxyls after the opening of the epoxide ring. The spectra revealed other interesting features of the reaction: upon irradiation, between 1760 cm^−1^ and 1700 cm^−1^ some weak peaks appeared, which could be attributed to the autooxidation of the residual double bonds in the aliphatic chain [5]. The intensity of the peaks at 2927 cm^−1^ and 2854 cm^−1^, and at 1600 cm^−1^ and 1583 cm^−1^ did not change; however, the peaks became slightly broader. The FT-IR spectra of the cured resins of this work were similar to those present in the literature [14].

The high degree of curing of the EC-5%PI resin was confirmed by measuring the insoluble content, which was in the 86–89 % range both in acetone and in toluene.

**Figure 8:**
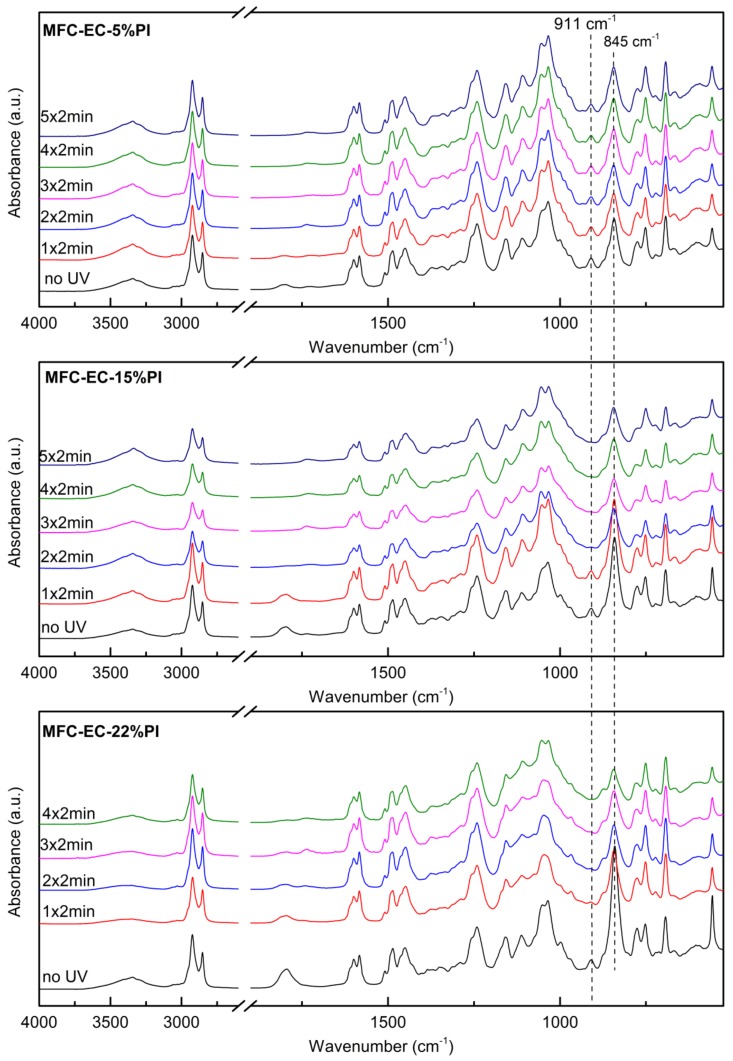
ATR FT-IR spectra composites MFC-EC-5%PI, MFC-EC-15%PI and MFC-EC-22%PI with different times of UV irradiation at 100 mW cm^−2^ (“n × 2 min” indicates that irradiation was carried out for n minutes on each side).

The photocuring of the composites upon exposure to UV radiation at 100 mW cm^−2^ for increasing time was followed by FT-IR analysis in ATR mode, as described in Section 3. The ATR FT-IR spectra of the composites with EC-5%PI, EC-15%PI and EC-22%PI resins before, and after different times of light exposure are shown in Figure 8. When the MFC mat was impregnated with the resins, the intensity of the O–H stretching signal diminished, and the characteristic peaks of the resins, as described in Section 2.1, appeared. When the 5% PI resin composites were irradiated, a moderate decrease of the epoxy signals was initially visible; however, after 2 min the conversion reaction did not seem to proceed further. When the composites with 15% and 22% PI resins were irradiated for increasing time, the same trends as for the resin alone were seen. The peak at 911 cm^−1^ diminished and eventually disappeared completely, after irradiating for more than 2 min per side, faster at the higher PI concentration. The photocuring was however slower than for the resin alone. The consumption of the photoinitiator was confirmed by the decrease of the peaks at 1800 cm^−1^ and 845 cm^−1^, which initial intensity increased with PI concentration in the resin. As the cationic curing is known to proceed also after the end of irradiation, partially cured samples with EC-5%PI and EC-15%PI were kept in the dark, and FT-IR spectra were taken again after 1 month. The MFC-EC-15%PI composite was eventually fully cured, while the MFC-EC-5%PI composite did not show a noticeable increase of the degree of curing, suggesting that for the latter all the available photoinitiator was consumed during the initial irradiation, and no further dark curing occurred.

### 3.2. Characterization

**Figure 9:**
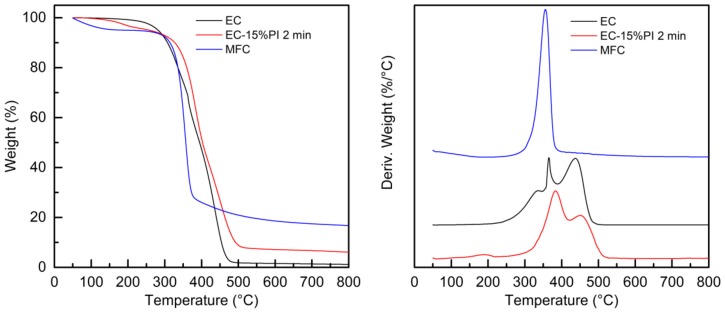
Weight (left), and first derivative (right) as function of temperature for the epoxidized cardanol resin EC, cured EC-15%PI resin, and dry MFC mat.

**Figure 10:**
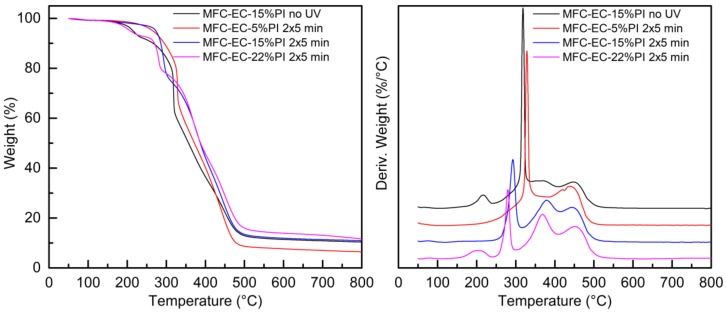
Weight (right) and derivative of the weight (left) as a function of temperature for uncured and cured composites.

The thermal stability of the dry MFC mat, of the EC monomer and of the fully cured EC-15%PI resin, as well as that of the uncured and cured composites was evaluated by thermogravimetric analysis in an inert atmosphere. The weight-loss curves, and their first derivatives, are reported in Figure 9 and Figure 10. The thermogravimetric measurements were performed after 1 month from curing.

The dry MFC mat degraded in one step at around 355 °C, with a residue of about 17% at 750 °C. The initial weight loss below 100 °C can be attributed to adsorbed humidity. The EC presented its first weight loss well above 200 °C; the derivative curve highlighted three different degradation events, with maximum degradation rates at about 335 °C, 365 °C and 440 °C. The residual weight was of about 1%. For the cured EC-15%PI resin only two main degradation steps were present, with maximum rates at 380 °C and 450 °C, and a small degradation step appeared at about 190 °C attributable to the degradation of the photoinitiator. The residue was above 6%, confirming that crosslinking took place. As a comparison, the same epoxidized monomer, crosslinked by thermal curing, the temperature at which a weight loss of 30% had occurred was reported to be in the 350–366 °C range [10,31], and its char at 600 °C between 2% and 6.8% [10]. The thermal stability of our resin cured by UV light is also comparable to that of a different EC resin cured by electron beam with hexafluorophosphate photoinitiator [12].

The uncured and cured composites showed complex weight profiles, which are reported in Figure 10, together with the corresponding first derivatives that enable better visualization of the different weight-loss events.

Although a detailed understanding of all the degradation mechanisms involved is beyond the scope of this work, some general considerations can be done. The small initial weight loss before 100 °C can be attributed to evaporation of residual solvent, as seen also in the DSC analysis. The second weight loss for the uncured composite, around 200 °C, can be attributed to loss of the photoinitiator; this weight-loss step was not observed for the cured composites with 5% and 15% photoinitiator, while it was present in the cured composite with 22% of photoinitiator, suggesting that only at the highest concentration not all the photoinitiator was consumed even after dark curing. The sharp weight loss present for the uncured composite with maximum rate at about 320 °C was attributed to the degradation of the cellulose microfibrils; for the composite with 5% photoinitiator after exposure to UV light this weight loss was found at about 330 °C. In both cases the degradation temperature is slightly lower than for the dry MFC mat. Remarkably, for the cured composites with 15% and 22% PI a dramatic shift of MFC degradation to lower temperatures (280–290 °C) is detected. This effect is attributed to the action of the cationic photoinitiator, which upon irradiation generates a strong Brønsted acid. In the composites, the acid, which is meant to promote the cationic polymerization of the epoxidized monomer, is supposed to attack also the cellulose microfibrils, hydrolyzing them. Moreover, this mechanism may explain why a higher amount of photoinitiator is required to cure the composites with respect to the neat resin. In the Supplementary Information (Figure S3) is reported the thermogravimetric analysis of MFC impregnated with only photoinitiator and irradiated, showing a highly reduced thermal stability. For the uncured composite, the first degradation step characteristic of the uncured resin was present, overlapping with the MFC degradation, and the second and third degradation steps were visible with maximum rates at 370 °C and 445 °C. A similar pattern was shown for the UV irradiated MFC-EC-5%PI composite, demonstrating that the crosslinking reaction did not advance for this composite. For the cured MFC-EC-15%PI and MFC-EC-22%PI composites the first degradation step of the resin was not present, while the second and third steps became more marked; particularly the second step, which appeared at 370–380 °C, showed an increased intensity, as seen for the cured EC-15%PI resin. The residual weights of 7–12% shown by these composites is compatible with a presence of 20–30 wt.% of MFC.

**Figure 11:**
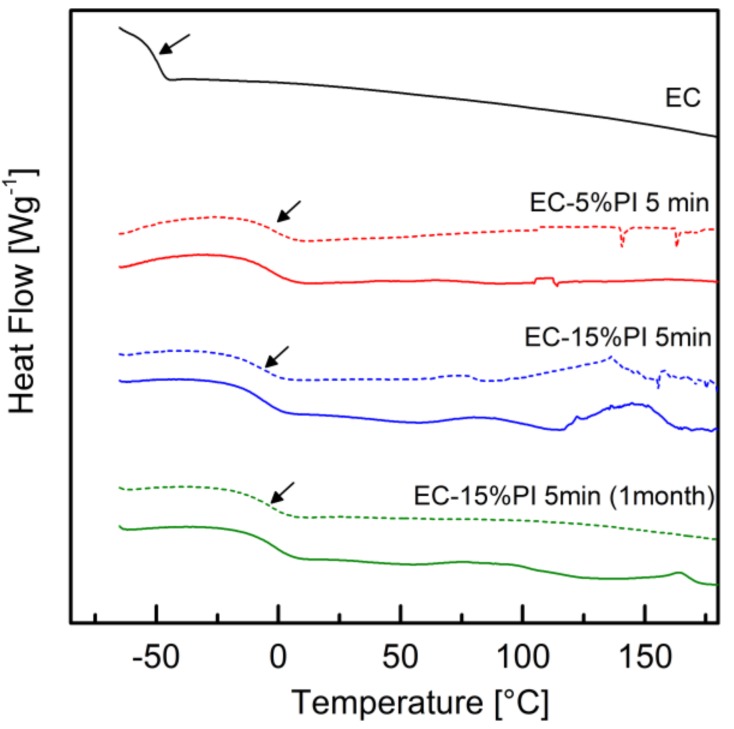
DSC thermograms of EC, of cured (5 min at 100 mW cm^−2^) EC-5%PI and of cured EC-15%PI (5 min at 100 mW cm^−2^) tested immediately and after one month from irradiation; full lines indicate the first heating scan, and dashed lines the second heating scan. The arrows indicate the glass transition region.

The EC monomer, as well as the EC-5%PI and EC-15%PI fully cured resins, were analyzed by DSC. The DSC thermograms are shown in Figure 11.

The curve of the uncured EC resin showed an inflection at −49 °C attributable to the glass transition. After curing, the glass transition temperature of the EC-5%PI resin increased to −4 °C; no other transitions could be remarked above this temperature. Furthermore, for both the uncured EC and the EC-5%PI cured resin no difference could be detected between the first and the second heating scan. On the other hand, for the EC-15%PI cured resin, a glass transition temperature of −7 °C was detected in the first heating scan, followed by two exothermic events that may indicate some residual reactivity. In the second heating scan the T_g_ increased to −3 °C, and the exothermic events were less intense. When this cured resin was tested one month after curing, the T_g_ was −3 °C both in the first and in the second heating scans; the exothermic events were very mild in the first heating scan, and disappeared in the second heating scan, indicating that the residual reactivity was almost completely suppressed. As a comparison, for EC thermally cured with amine hardeners, glass transition temperatures ranging from 9 °C to 158 °C were reported, depending on the type and amount of hardener used [10,31]. Furthermore, the maximum T_g_ obtained for an EC cured by electron beam with an hexafluorophosphate photoinitiator was −2.9 °C by DSC, while by DMA it was found to be around 13 °C; with an hexafluoroantimonate photoinitiator higher T_g_ were obtained [13]. The glass transition temperatures shown by a cardanol epoxy prepolymer cured by UVC with hexafluorophosphate photoinitiators combined with photosensitizers were around 25−30 °C by DSC and around 20 °C by DMA [14]. These higher results however must take into account the effect of the prepolymerization of the cardanol epoxy prepolymer on T_g_.

**Figure 12:**
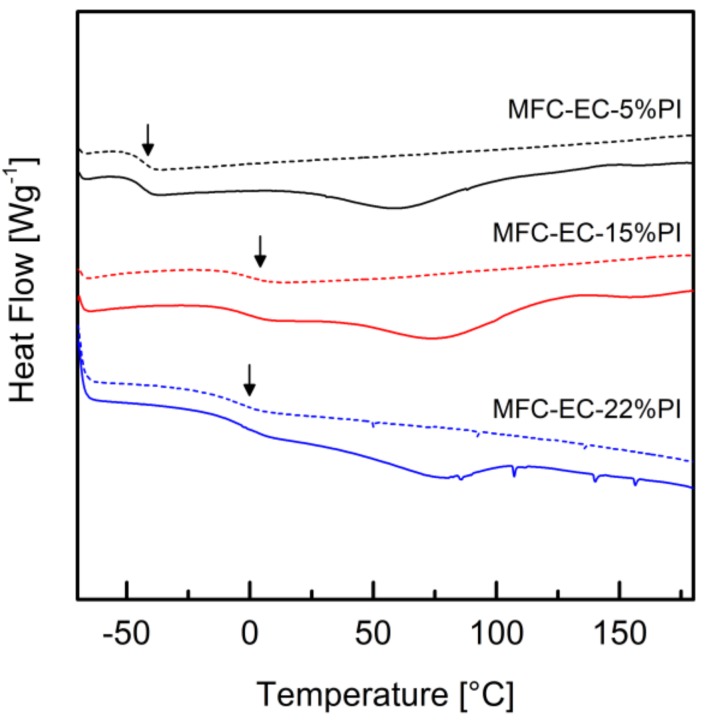
DSC thermograms of the cured composites with different photoinitiator concentrations. Full lines are for the first heating run and dashed lines for the second heating run in the DSC. The arrows indicate the glass transition region.

The DSC thermograms of the composites are shown in Figure 12. The thermogram of the uncured MFC-EC-5%PI composite showed a step corresponding to the glass transition temperature of the uncured resin at −48 °C. The thermogram of the MFC-EC-5%PI composite irradiated for 5 min showed a T_g_ at −44 °C, confirming that negligible cure occurred. For the MFC-EC-15%PI and the MFC-EC-22%PI composites irradiated for 5 min the T_g_ was in the −3 °C to 1 °C range, similarly to what shown by the fully cured resin. The first heating run of all composites, before and after curing, also showed an endothermic peak around 60–75 °C, which disappeared in the second heating run, and which can be attributed to the evaporation of residual solvent.

**Figure 13:**
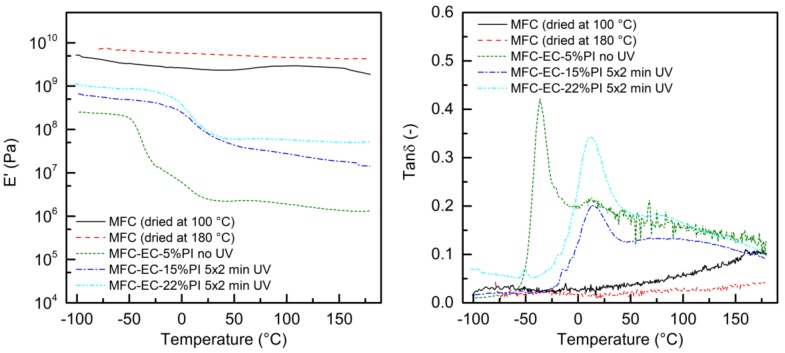
Storage modulus E’ and loss tangent (tan δ) as a function of temperature for uncured and cured composites, and of the MFC mat dried at 100 °C and 180 °C.

The storage modulus (E’) and the loss tangent (tan δ) as a function of temperature, measured for the uncured and cured composites, and for the dry MFC mat, are reported in Figure 13. The dry MFC mat did not show prominent thermal transitions in the explored temperature range, resulting in almost flat E’ and tan δ curves. The E’ was of about 10^9^ Pa in the entire temperature range. At around 50 °C, a dip in the E’ curve, which may be due to the evaporation of residual solvent, appeared for the mat dried at 100 °C, but was not present for the mat dried at 180 °C. For the composites, the thermal transitions of the resin were detected during the temperature scan. When no UV-curing was performed on the composites, the E’ before glass transition was around 10^8^ Pa, and a first large peak in the tan δ curve, related to the glass transition of the resin, was present with a peak temperature that varied in the −50 °C to −35 °C range, depending on the tested specimen. A smaller peak appeared in some cases at 10 °C. This suggests that although care was taken to shorten the time between specimen preparation and testing, keeping the sample in the dark and refrigerated, some curing of the resin may have happened even without UV irradiation, as also suggested by TGA. When the MFC-EC-15%PI and MFC-EC-22%PI composites were cured for 5 min per side at 100 mW cm^−2^ the peak of tan δ associated with the glass transition temperature shifted to about 12 °C. A very small peak was still visible at −52 °C, possibly due to some uncured resin residues. Above the glass transition temperature, the E’ dropped of about two orders of magnitude for uncured composites, while the decrease was smaller for the cured composites. The T_g_ obtained by DMA for the fully cured composite was similar to that obtained by DMA for the electron beam cured resin reported in [13], which also presented a similar difference between the T_g_ detected by DSC and that measured by DMA as that reported here.

As mentioned above, MFC are hydrophilic and sensitive to water. As expected, the contact angle on the dry MFC mat was lower than 10° as soon as the drop touched the surface, while after a few seconds the drop was absorbed by the mat. The wetting by water of the MFC-EC-22%PI cured composites was hindered: the water contact angle was found to be in the 75° to 85° range, at different locations of a same specimen. This may be due to the microfibrils being covered by a thinner or thicker layer of resin at different locations of the surface, as the resin may fill the valleys of the rough MFC mat surface, as suggested also from the optical micrographs. Although the value is lower than 90° and the materials cannot be defined hydrophobic, the wettability of the composite is remarkably poor. In agreement with this result, the water uptake of the cured MFC-EC-15%PI composite after four weeks of immersion was found to be only 8%, so that the composites can be considered water resistant.

## 4. Conclusions

In this work we demonstrated that EC can be successfully cured by UV radiation, with a cationic photoinitiator, obtaining a transparent film at the rubbery state with a T_g_ below room temperature.

The resin was compatible with MFCs, the photocuring reaction of the composites proceeded to completion, although with a very high percentage of photoinitiator. The obtained films were transparent, rubbery, flexible, and water repellent. To fully exploit these materials, it might be interesting to increase the glass transition temperature, which can be easily done by copolymerization with other biobased polyfunctional epoxide resins.

## Supplementary Materials

The following are available online: Figure S1: MFC dry mat obtained by solvent exchange followed by evaporation of the solvent in air (left), and its surface observed with a stereo microscope in reflection mode. Figure S2: Dry MFC mat (left) and MFC mat impregnated with photoinitiator, irradiated for 5 min at 100 mW cm^−2^ (right). Figure S3: Thermogravimetric analysis of MFC mat impregnated with photoinitiator, irradiated for 5 min at 100 mW cm^−2^: weight profile and its first derivative as a function of temperature.
