# Supplementary material for: Photocuring of Epoxidized Cardanol for Biobased Composites with Microfibrillated Cellulose
Source: Molecules. 2019 Oct 25;24(21):3858. doi: 10.3390/molecules24213858 (PMC6864556; doi:10.3390/molecules24213858)
Supplement: Supplementary file 1 [file molecules-24-03858-s001.pdf]

Photocuring of epoxidized cardanol for biobased composites with microfibrillated cellulose.

Sara Dalle Vacche [1,*], Alessandra Vitale[1] and Roberta Bongiovanni [1]

[1] Department of Applied Science and Technology, Politecnico di Torino, Corso Duca degli Abruzzi 24, 10129 Torino, Italy; (A.V.) alessandra.vitale@polito.it; (R.B.) roberta.bongiovanni@polito.it

* Correspondence: sara.dallevacche@polito.it; Tel.: +39-011-090-4565

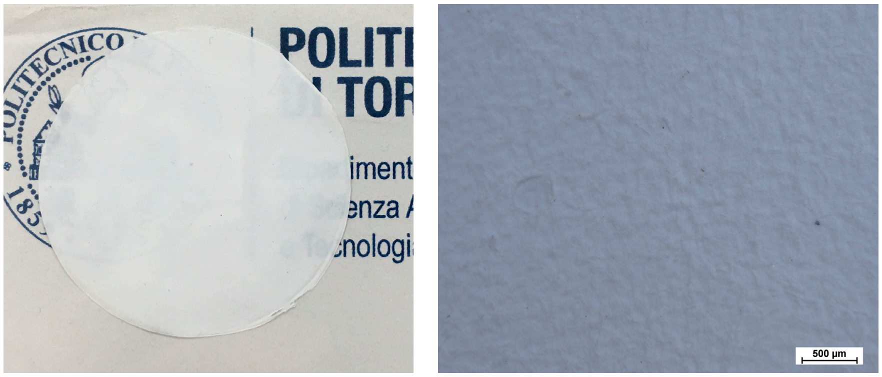

Figure S1. MFC dry mat obtained by solvent exchange followed by evaporation of the solvent in air (left), and its surface observed with a stereo microscope in reflection mode.

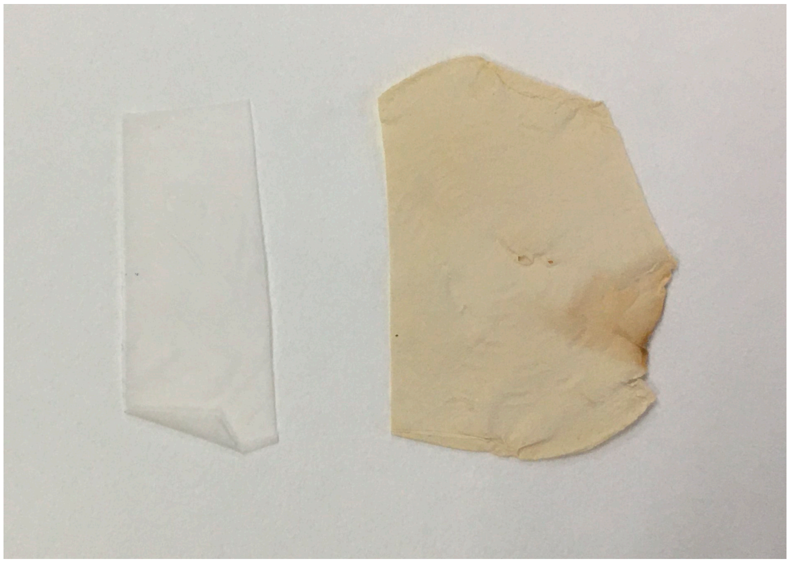

Figure S2: Dry MFC mat (left) and MFC mat impregnated with photoinitiator, irradiated for 5 minutes at 100 mW cm^{-2} (right).

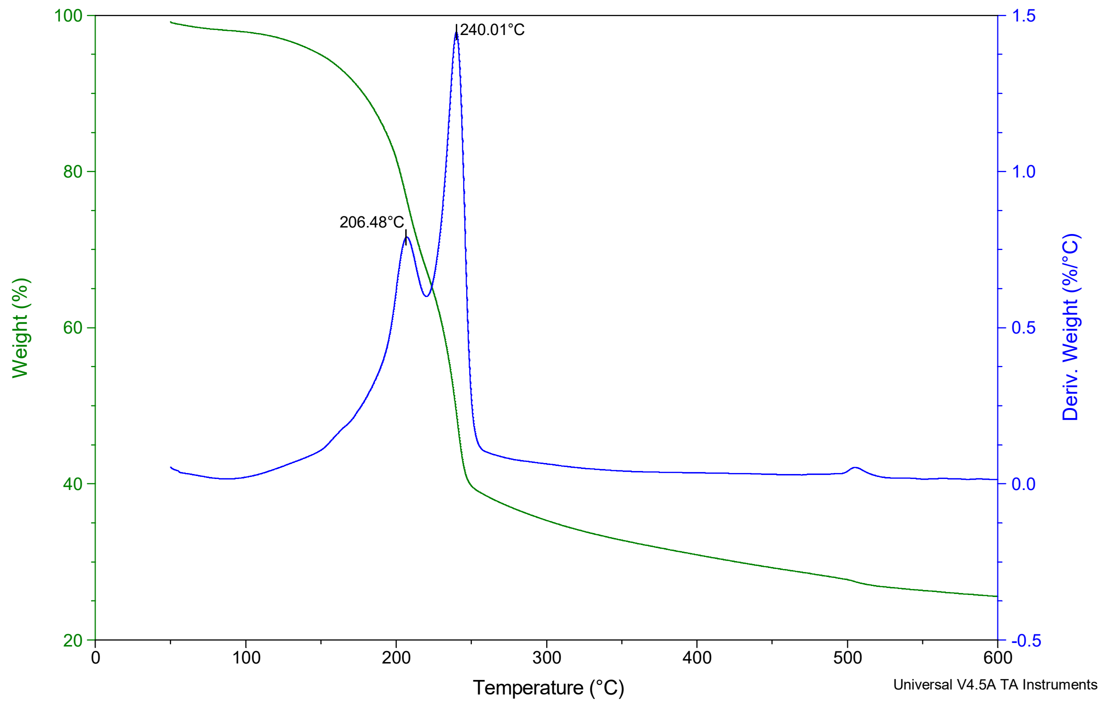

Figure S3. Thermogravimetric analysis of MFC mat impregnated with photoinitiator, irradiated for 5 minutes at 100 mW cm^{-2}: weight profile and its first derivative as a function of temperature.
